# Supplementary material for: A Surprising Diversity of Xyloglucan Endotransglucosylase/Hydrolase in Wheat: New in Sight to the Roles in Drought Tolerance
Source: Int J Mol Sci. 2023 Jun 8;24(12):9886. doi: 10.3390/ijms24129886 (PMC10297901; doi:10.3390/ijms24129886)
Supplement: Supplementary file 1 [file ijms-24-09886-s001.zip › Supplementary Files S1 Structural alignment of 71 TaXTH proteins based on amino acid sequences.pdf]

```
1un1
                                      1        10        20        30
1un1          ........................MAAAYPWTLFLGMLVMVSGTMGAALR...KPVDVAF
TaXTH1        ...............................MARPSLSLHLC...LAVLAMAAAASEAGF
TaXTH2        ...............................MARPSLSLHLC...LAVLALAAAASEAGF
TaXTH3        ...............................MARPSISLHLC...LAVLAMAAAASEAGF
TaXTH4        ..............................MAARSSALLAVL..VLLAVSFLA.GAKADF
TaXTH5        ....................................MRTVAIGILA.MACLVAVARGGNF
TaXTH6.1a     ....................MAAASALLMA.....ALAVFAAAAAAALDTS.....PVPF
TaXTH6.1d     ....................MAAASALLMA.....ALAVFATAAAAALDTS.....PVPF
TaXTH6.2a     ...............................MARPSLSLHLC...LAVLALAAAASEAGF
TaXTH6.2d     ...............................MARPSLSLHLC...LAVLAMAAAASEAGF
TaXTH7.1a     ..........................MGRLARRARLLASLVEAFYIILAVSPVAS...DM
TaXTH7.1b     ..........................MGRLARRAYLLGSLIEAFYLILAVSPVAS...DM
TaXTH7.1d     ............................................................
TaXTH7.2a     ..........................MARGARRARLLAS.LQAIYLILAVSQVAG...DM
TaXTH7.2b     ................................MGKPGA.LVPVIALAFALVLGLVSGGNF
TaXTH7.2d     ........................MGRPCVGALLACAAVAASCCGCFQFQGAAAAATPSF
TaXTH7.3a     MLAQFHPSRANIHSQSTRTRIENMSSPLLVAMAAAAIVVACC...LAACPVGAGASAGDF
TaXTH7.3b     .......................MSRP.LLAMAAAAIVVACC...FAACPVGAGASAGGF
TaXTH7.3d     .......................MSSPLLVAMAAAAIVVVCC...FAACPVSAGASAGGF
TaXTH7.4a     ............................MAVSSKQAYACVVLVVLCIAARTAAAWG..RI
TaXTH7.4b     ............................MALSSKQVHACVVLVVLCIAARTAAAWG..RI
TaXTH7.4d     ............................MAVSSKQAHACVVLVVLCIAARTAAAWG..RI
TaXTH7.5a     ...........................MALARERLLASLSALALMIAAWASPVAGG..HP
TaXTH7.5b     ...........................MALATEGLLASLSALALMIAAWASPVAGG..RP
TaXTH7.5d     ...........................MALARERLLASLSALALMFAAWASPVAGG..RP
TaXTH7.6a     ................................MESSRR.ALLLVAMA.ATAIGLAS.ASF
TaXTH7.6d     ................................MESSRR.ALLLVAVA.AAAIGLAS.ASF
TaXTH7.7d     ................................MESSRR.ALLLVAVA.ATAIGLAG.ASF
TaXTH8.1a     ...................MTFSL.KPWALLLVLLLFVGLSLG...........GPATVF
TaXTH8.1b     ...............................MAR.HFLAVLA...VALALLQVASAKSWL
TaXTH8.2b     ...............................MAR.HFLAMLA...VVLALSQATSAKPWL
TaXTH8.3b     ...................MTSSL.KPWALLLVLLLFAGLSLG...........APATVF
TaXTH8.4b     ...................MTSSL.QPWALLLVLFLSNVLSLG...........APATVF
TaXTH9.1a     ...................MASRLLLAVLLASSAWVAASAGAGDEVMVPRP...AAALTF
TaXTH9.1b     ...................MAGRFLLAVLLASSSWVAASAGAADEVMVPRP...AAALTF
TaXTH9.1d     ........................MKATAGALLAVVAAVLLRGVAAAPPR...KPVDVPF
TaXTH9.2a     ........................MKATAGALLAVVAAVLLRGVAAAPPR...KPVDVPF
TaXTH9.2b     ......MMTMMMQIRPHIGISRLMVVVGAVVLLLLLQGRPAEAQPSPGYYPSSKVSSMPF
TaXTH9.2d     ...................MACRFLLAVLLASSAWVAASAGAADEVMVPRPAAAAAALTF
TaXTH9.3a     ...................MAAAAVTLLLLFA..SCMHAAVFCAAAPFDEVP....TVTF
TaXTH9.3d     ......MMTMMVQIRPHIAIPHLMVVVGAVMLLLLQQGRSAEAQPSPGYYPSSKVSSMPF
TaXTH9.4a     ...............................MARWAAVCAVA...VALALLAASSAAAWL
TaXTH10.1a    .......MTMMMQIRPHIAISNLMVVVGAVMLLLLQQGRWAEAQPSPGYYPSSKVSSMPF
TaXTH10.1b    .............................MTMLRAVSVTVGV...AVALLAAATAAESWL
TaXTH10.1d    .............................MAMLRAVSVTVGV......LLAAATAADSWL
TaXTH11.1a    ...................MSRLASALLALAT..AAAVAVAVAARPAVDVTA....TVAF
TaXTH11.1b    ...................MSRLASALLALAT..VAAVAVAV..RPTVDVTA....TVAF
TaXTH11.1d    .........................MASSLPSSSCWHSVLLVAMLLVVVMDQMAMA..YM
TaXTH11.2a    ...................MAMKAGFLAA....AAACVCLAAAAA.AFDVP.....TVAF
TaXTH11.2b    ...................MVMKARFLAASLAVAATCVCLAAAAASAFDVP.....TMAF
TaXTH11.3a    .........................MASSLPSSSCWHSMVLVAMLVVVVVDQMAMA..YM
TaXTH11.3b    .........................MASSLPSSSCWHSIVLVAMLLVLVMDQMAMA..YM
TaXTH11.4b    ..............................MAARSSALLAVLAVALAAVSFLLTGARADF
TaXTH12.1a    .............................MAMPRAVSLSVAV......LLALLASAESWL
TaXTH12.1b    ...................MASSVRQPWPLLLIVLLP...SL............ATATVF
TaXTH12.1d    ............................................................
TaXTH12.2a    ...............................MASRAAVSA......ALALLVAS.ASAWL
TaXTH12.2b    ................................MGMSMRALLAVVAFAAVLRLGRVS.ANF
TaXTH12.2d    ...............................MASPAAVSAVA...VALALLVAS.ADAWL
TaXTH12.3a    ...................MASSVRQPWHLLLIVLLP...SL............ATATVF
TaXTH12.3b    ...................MASLSMLPAMALLLLAMAVPSSDAQPSPGYYPSSRFRPMAF
TaXTH12.3d    ...................MASSVRQPWQLLLIVLLP...SL............AAATVF
TaXTH12.4a    ................................MGRSMRGLLAVVAFAAVLRLGLVG.ANF
TaXTH12.4b    ....................................MAVSVLAILL.ASCALAAAS...F
TaXTH12.4d    ................................MGRSMGGLLAVVAFAAVLRLGLVG.ANF
TaXTH12.5a    ....................................MAVSVLAILL.ASCALAAAS...F
TaXTH12.5d    ...................MASLSMLPAMALLLLAMAVASSDAQPSPGYYPSSRFRPVAF
TaXTH12.6a    ....................................MAVSVLAILL.ASFALAAAS...F
TaXTH12.6d    .................................MAVSSVL..AILLDASCALASAT...F
TaXTH12.7a    ............................MASSPRRTVVCSVLPLLLLLAGAARAAG..NF
TaXTH12.7d    ....................................MAVSVLAILL.ASCALAVAS...F
TaXTH12.8d    .................................MARMAVLGLAILLAASCALAAAS...F
```

```
            α1    β1       η1        β2                        η2
1un1
                      40              50
1un1        GRNYVPTWAFD.....HIKYFN...................................GGN
TaXTH1      YDQFDVVGSGN.NVRVNDDG.....................................LAQ
TaXTH2      YDQFDVVGSGS.NVRVNDDG.....................................LAQ
TaXTH3      YDQFDVVGSGN.NVRVNDDG.....................................LAQ
TaXTH4      DDQFEVIGDRD.HIGYRDDGKD...................................KGQ
TaXTH5      FQDSEMTWGDG....RGKVVD....................................GGR
TaXTH6.1a   DAGYAPLFGGDNLVRSADGRSVTLKLDRYTSCGGDNLVPFDAGYAPLFGGDNLVRSADGR
TaXTH6.1d   DAGYAPLFGGDNLVRSVDGR........................................
TaXTH6.2a   YDQFDVVGSGS.NVRVNDDG.....................................LAQ
TaXTH6.2d   YDQFDVVGSGN.NVRVNDDG.....................................LAQ
TaXTH7.1a   TDSLDLLWGNTQ...VEYDSS....................................GRQ
TaXTH7.1b   TDSLDLLWGDTK...VEYDSG....................................GRQ
TaXTH7.1d   ............................................................
TaXTH7.2a   TDSLDLLWGNTQ...VEYDSS....................................GRQ
TaXTH7.2b   YDECDATWEPQ.....NCWTYD...................................GGN
TaXTH7.2d   GDNFDITGAED.....HVKTSP...................................DGQ
TaXTH7.3a   YDNFVVKWGTDPDPDRRVEIVD...................................GGR
TaXTH7.3b   YDNFVVKWGTDPDPDRRVEIVD...................................GGR
TaXTH7.3d   YDNFVVKWGTDPDPDRRVEIVD...................................GGR
TaXTH7.4a   DDRLEVNWGHGS...HGTVSA....................................DGQ
TaXTH7.4b   DDGLEVNWGRGS...HGTVSA....................................DGQ
TaXTH7.4d   DDRLEVNWGRGS...HGTVSA....................................DGQ
TaXTH7.5a   ADQLQILWGQTK...VLSDGN....................................GDQ
TaXTH7.5b   ADQLQILWGQTQ...VLNDGN....................................GDQ
TaXTH7.5d   ADQLQILWGQTK...VLNDGN....................................GDQ
TaXTH7.6a   RDNCDIKWNPE.....NAAFSD...................................DGH
TaXTH7.6d   RDNCDIKWNPE.....NAAFSD...................................DGH
TaXTH7.7d   RDNCDIKWNPE.....NAAFSD...................................DGH
TaXTH8.1a   NENFVPVWCADGYHLANHGT........................................
TaXTH8.1b   DKRFNTDG....TVRTGYDAS....................................GRE
TaXTH8.2b   DDKFNTDG....NVRTGYDAS....................................GEQ
TaXTH8.3b   NENFVPVWGADGYHLANHGT........................................
TaXTH8.4b   NENFVPVWGADGYHLANQGT........................................
TaXTH9.1a   GEGYTQLFGDSNLRLHGDGK........................................
TaXTH9.1b   GEGYTQLFGDSNLRLHGDGK........................................
TaXTH9.1d   EKNYVPTWAQD.....HIHYVN...................................GGR
TaXTH9.2a   DKNYVPTWAQD.....HIHYVN...................................GGR
TaXTH9.2b   SQWYTNLWGAQHQSLSPDQT........................................
TaXTH9.2d   GEGYTQLFGDSNLRLHGDGK........................................
TaXTH9.3a   DEGFAPLFGESNMGRSSGGD........................................
TaXTH9.3d   SQWYSNLWGAQHQSLSPDQT........................................
TaXTH9.4a   HEEFTTEG....NVRAGYDAR....................................GQQ
TaXTH10.1a  SQWYSNLWGAQHQSLSPDQT........................................
TaXTH10.1b  YEEFTTDG....NVRADYNAQ....................................GQQ
TaXTH10.1d  YEEFTTDG....NVRADYNAQ....................................GQQ
TaXTH11.1a  GEGYTPLFGFDNILRSADDR........................................
TaXTH11.1b  GEGYTPLFGFDNILRSADDR........................................
TaXTH11.1d  YDDIEIVWGDDHSFFYMDDAG....................................DDE
TaXTH11.2a  EEGFSPLFGDGNLVRARDDR........................................
TaXTH11.2b  EEGFSPLFGDGNLVRARDDR........................................
TaXTH11.3a  YDDIEIVWGDDHSFFYMDDAG....................................DDE
TaXTH11.3b  YDDIEIVWGDDHSFFYMDDAG....................................DDE
TaXTH11.4b  DDQFEAIGDRD.HIGYRDDGED...................................KGQ
TaXTH12.1a  YEEFTTDG....NVRADYNAQ....................................GQQ
TaXTH12.1b  DDNYVPSWGADGYHLVDQGT........................................
TaXTH12.1d  ..........................................................MK
TaXTH12.2a  HEEFTTEG....NVRAGYDAR....................................GQQ
TaXTH12.2b  ADLCDITWEPQ.....NAAMTD...................................GGE
TaXTH12.2d  HEEFDTEG....NVRAGYDAR....................................GQQ
TaXTH12.3a  DNNYVPSWGADGYHLVDQGT........................................
TaXTH12.3b  NRGYRNKWGPQHQTLSGDHS........................................
TaXTH12.3d  DDNYVPSWGADGYHLVDQGT........................................
TaXTH12.4a  ADLCDITWEPQ.....NAAMTD...................................GGE
TaXTH12.4b  DKEFDVTWGDG....RGKILN....................................NGQ
TaXTH12.4d  ADLCDITWEPQ.....NAAMTD...................................GGE
TaXTH12.5a  DKEFDVTWGDG....RGKILD....................................NGQ
TaXTH12.5d  NRGYRNKWGPQHQTLSGDHS........................................
TaXTH12.6a  DKEFDITWGDG....RGKILN....................................NGQ
TaXTH12.6d  DKEFDITWGDG....RGKIMN....................................NGQ
TaXTH12.7a  YQDVDITWGDG....RGKIGG....................................GGD
TaXTH12.7d  DKEFDITWGDG....RGKILN....................................NGQ
TaXTH12.8d  DKEFDITWGDG....RGKILN....................................NGQ
```

```
                 β3          β4           β5               β6
1un1                                                  TT
                       60         70         80         90        100
1un1          EIQLHLDKYTG.TGFQSKGSYLFGHFSMQMKLVPGDSAGTVTAFYLSS.....QNSEHDE
TaXTH1        QVALTLDQSNGGSGFSSKDKYLYGEFSVQMKLIGGNSAGTVTSFYLTSGE....GDGHDE
TaXTH2        QVALTLDQGNGGSGFSSKDKYLYGEFSVQMKLIGGNSAGTVTSFYLTSGE....GDGHDE
TaXTH3        QVALTLDQGNGGSGFSSKDKYLYGEFSVQMKLIGGNSAGTVTSFYLTSGE....GDGHDE
TaXTH4        EFSLELDQ.ESGSGFKSKAKYLFGEFEVKMKLVDGNSAGTVTSFYLTSGE....SATHDE
TaXTH5        GLDLTLDKTSG.SGFQSKTEYLFGKIDMQIKLVPGNSAGTVTTFYLSSQG.....TAHDE
TaXTH6.1a     SVTLKLDRYTG.SGFISKSAYRHGFFGASIKLPADYTAGVVVAFYLSNWD.EYPK.NHDE
TaXTH6.1d     SVTLKLDRYTG.SGFISKSAYRHGFFGASIKLPADYTAGVVVAFYLSNWD.EYPK.NHDE
TaXTH6.2a     QVALTLDQGNGGSGFSSKDKYLYGEFSVQMKLIGGNSAGTVTSFYLTSGE....GDGHDE
TaXTH6.2d     QVALTLDQSNGGSGFSSKDKYLYGEFSVQMKLIGGNSAGTVTSFYLTSGE....GDGHDE
TaXTH7.1a     TVSLSLDRWTT.SAFRSKSMHLFGRFDMDIKLVPRDSAGTITTLYMLTEG...PWEEHDE
TaXTH7.1b     TVSLSLDRWMT.SVFRSKSKHLFGRFDMDIKLVPGDSAGTVTTLYMLTEG...PWEEHDE
TaXTH7.1d     ...........................MDIKLVPRDSAGTITTLYMLTEG...PWEEHDE
TaXTH7.2a     TVSLSLDRWTT.SAFRSKSTHLFGRFDMDIKLVPRDSAGTITTLYMLTEG...AWEEHDE
TaXTH7.2b     SLSLALVSNSSGSMIRSKRQFIYGTVSTMIQLVKGDSAGTVTTYYTSSVG.....DDHDE
TaXTH7.2d     TWYLSLDNKTG.VGFQTKQKYLFGWFSMKLKLVGNDSAGVVTAYYMCSDLD..AAPERDE
TaXTH7.3a     LVTLTLNNVSG.AGFQSRDAFLFGEFTMEMKLVPGDSAGTVTTFYLTSKDPTAAGDGHDE
TaXTH7.3b     LVTITLNNVSG.AGFQSRDAFLFGEFTMEMKLVPGDSAGTVTTFYLTSKDPTAVGDGHDE
TaXTH7.3d     LVTLTLNNVSG.AGFQSRDAFLFGEFTMEMKLVPGDSAGTVTTFYLTSKDPTAAGDGHDE
TaXTH7.4a     VISLSLDRNSG.SGFRSRDTYLYARIDLQIKLAPGNSAGTVTTCYFLSEG...SWANHDE
TaXTH7.4b     VVSLSLDRNSG.SGFRSKDTYLYARIDLQIKLAPGNSAGTVTTCYFLSEG...SWANHDE
TaXTH7.4d     VISLSLDRNSG.SGFRSRDTYLYARIDLQIKLAPGNSAGTVTTCYFLSEG...SWANHDE
TaXTH7.5a     TIALMLDHAMG.SAFKSKTSYLFARIDVDIKLIPRNSAGTVTTIYMISEK...DWKTHDE
TaXTH7.5b     TIELMLDHAMG.SAFKSKTSYLFARIDVDIKLIPRNSAGTVTTIYMISEK...DWKTHDE
TaXTH7.5d     TIALMLDHAMG.SAFKSKTSYLFARIDVDIKLIPRNSAGTVTTIYMISEK...DWKTHDE
TaXTH7.6a     GLTMSLKSNSSGCLLQTKKQFIYGSVSTLIKLVPGNSAGTVTTYYTSSVG.....ADHDE
TaXTH7.6d     GLTMSLKSNSSGCLLQTKKQFIYGSVSTLIKLVPGNSAGTVTTYYTSSVG.....ADHDE
TaXTH7.7d     GLTMSLKSNSSGCLLQTKKQFIYGSVSTLIKLVPGNSAGTVTTYYTSSVG.....ADHDE
TaXTH8.1a     QVSLTMDRNSG.AGFSSKMMYGSGLFHMRIKIPAGYTAGVVTAFYLTTQP.EYG..DHDE
TaXTH8.1b     VVMLNLNQQSGAAGFNSKEQFLYGEFSIQMKLIPGNSAGTVSCFYLSSGD....DEWRDE
TaXTH8.2b     VVTLSLDQHSG.AGFNSDEQYLYGEFSIQMKLIQGNSAGTVSCFYLSSGD....GDGHDE
TaXTH8.3b     QVSLIMDRNSG.AGFSSKMMYGSGLFHMRIKIPAGYTAGVVTAFYLTTQP.EYG..DHDE
TaXTH8.4b     QVSLTMDRNSG.AGFRSKMMYGSGLFHMRIKLPAGYTAGVVTAFYLTTEP.DYG..DHDE
TaXTH9.1a     RVHISLDERTG.SGFASQGAYLHGLFSASIKLPSDYAAGVVVAFYMSNGD.VYEK.THDE
TaXTH9.1b     RVHISLDERTG.SGFASQGAYLHGFFSASIKLPSDYAAGVVVAFYMSNGD.VYEK.THDE
TaXTH9.1d     EVQLSLDKTTG.TGFQTRGSYLFGHFSMHIKLVGGDSAGTVTAFYLSS.....QNSEHDE
TaXTH9.2a     EVQLSLDKTTG.TGFQTRGSYLFGHFSMHIKLVGGDSAGTVTAFYLSS.....QNSEHDE
TaXTH9.2b     SLTLWMDRSSG.SGFKSKRAYRNGYFGVSMKVQPGYTAGVNTAFYLSNNE.VYPG.YHDE
TaXTH9.2d     RVHISLDERTG.SGFASQGAYLHGFFSASIKLPSDYAAGVVVAFYMSNGD.VYEK.THDE
TaXTH9.3a     AVSITLDRSTG.SGFISKRYYHHGLFSADVKLPAGHTAGVVVAFYLSNGD.VFEA.THDE
TaXTH9.3d     ALTLWMDRSSG.SGFKSKGAYLNGYFGVSMKVQPGYTAGVNTAFYLSNNE.VYPG.YHDE
TaXTH9.4a     VASIVLDRQSGG.AFRSRRSYLYGQFSVQIKLVPGNSAGTVASFYLSSGD....GPGHDE
TaXTH10.1a    SLTLWMDRSSG.SGFKSKRAYRNGYFGVSMKVQPGYTAGVNTAFYLSNNE.AYPG.YHDE
TaXTH10.1b    VASLMLTQQSGGGAFGSRQKYLYGEFSIQMKLVPGNSAGTVTSFYLSSGD....GPGHDE
TaXTH10.1d    VASLMLTQQSGGGAFGSRQKYLYGEFSIQMKLVPGNSAGTVTSFYLSSGD....GPGHDE
TaXTH11.1a    TVSLLLDRTTG.SGFISSAMYQHGFFSASIKLPSDYTAGVVVAFYTSNGD.VFPK.THDE
TaXTH11.1b    TVSLLLDRSTG.SGFISSAMYEHGFFSASIKLPSDYTAGVVVAFYTSNGD.VFPK.THDE
TaXTH11.1d    ILALCLDQTHG.SGFHSKEAFLYARFDVDLMLVPDNSAGTVTTLYLMPEDV..PWDYHDE
TaXTH11.2a    AARLLLDRRSG.SGFISSDYYLHGFFSASIKLPRDYTAGVVVAFYLSNGD.VYEK.THDE
TaXTH11.2b    AARLLLDRRSG.SGFISSDYYLHGFFGASIKLPRDYTAGVVVAFYLSNGD.VYEK.THDE
TaXTH11.3a    ILALCLDQTHG.SGFHSKEAYLYARFDVDLMLVPDNSAGTVTTLYLMPEDV..PWDYHDE
TaXTH11.3b    VLALCLDQTHG.SGFHSKEAYLYARFDVDLMLVPDNSAGTVTTLYLMPEDV..PWDYHDE
TaXTH11.4b    EFSLELDQ.ESGSGFKSKAKYLFGEFEVKMKLVDGNSAGTVTSFYLTSGE....SATHDE
TaXTH12.1a    VASLVLTQQSGGGAFGSRQKYLYGQFSIQMKLVPGNSAGTVTSFYLSSGD....GPGHDE
TaXTH12.1b    ETRLTMDRTSG.AGFRSRSTYGSGFFHMRIKVPGGYTAGVITAFYLASEA.PYDGSDRDE
TaXTH12.1d    DKVLILTQQSGGGAFGSRRKYLHGEFSIQMKLVPGNSAGSVTSFYLSSGD....GPGHDE
TaXTH12.2a    VASIVLDRQSGG.AFRSRRRYLYGQFSVQIKLVPGNSAGTVASFYLSSGE....GPGHDE
TaXTH12.2b    HLTLSLVSNISGSMLRTKKTFIYGSISTLIKLVKGNSAGTVTTYYTSSVG.....DDHDE
TaXTH12.2d    VASIVLDRQSGG.AFRSRRSYLYGQFSVQIKLVPGNSAGTVASFYLSSGD....GPRHDE
TaXTH12.3a    ETRLTMDRTSG.AGFRSRSTYGSGFFRMRIKVPGGYTAGVVTAFYLASEA.PYDGGDRDE
TaXTH12.3b    AVTIWLDKTCG.SGFKSKHAYRNGYFATRIKLPAGYTAGTNTAFYLSNNE.AHPG.FHDE
TaXTH12.3d    ETRLTMDRTSG.AGFRSRSTYGSGFFHMRIKVPGGYTAGVITAFYLASEA.PYDGSDRDE
TaXTH12.4a    HLTLSLVSNISGSMLRTKKTFIYGSISTLIKLVKGNSAGTVTTYYTSSVG.....DDHDE
TaXTH12.4b    LLTLGLDKISG.SGFQSKHEYLYGKIDMQLKLVSGNSAGTVTAYYLSSQG.....PTHDE
TaXTH12.4d    HLTLSLVSNISGSMLRTKKTFIYGSISTLIKLVKGNSAGTVTTYYTSSVG.....DDHDE
TaXTH12.5a    LLTLGLDKVSG.SGFQSKHEYLYGKIDMQLKLVPGNSAGTVTAYYLSSQG.....PTHDE
TaXTH12.5d    AITIWLDKTCG.SGFKSKHAYRNGYFATRIKLPAGYTAGTNTAFYLSNNE.AHPG.FHDE
TaXTH12.6a    LLTLGLDKVSG.SGFQSKHEYLFGKIDMQLKLVPGNSAGTVTAYYLSSQG.....PTHDE
TaXTH12.6d    LLTLGLDKVSG.SGFQSKHEYLYGKIDMQLKLVPGNSAGTVTAYYLSSQG.....PTHDE
TaXTH12.7a    VLTLSLDRASG.SGFQSKNQYLYGRFDMQIKLVPGDSAGTVATFYLSSQG.....SAHDE
TaXTH12.7d    VLTLGLDKVSG.SGFQSKHEYLFGKIDMQLKLVPGNSAGTVTAYYLSSQG.....PTHDE
TaXTH12.8d    LLTLGLDKVSG.SGFQSKHEYLFGKIDMQLKLVPGNSAGTVTAYYLSSQG.....PTHDE
```

```
          β7              β8               β9          β10
1un1      ──────►   TT    ──────────► TT           ──►          ──────►
          110       120       130                  140          150
1un1      IDFEFLGNRTG..QPYILQTNVFTGGK......GDREQRIYLWFDPTKE.FHYYSVLWNM
TaXTH1    IDIEFMGNLSG..DPYVMNTNVWASGD......GKKEHQFYLWFDPTAD.FHTYKIVWNP
TaXTH2    IDIEFMGNLSG..DPYVMNTNVWASGD......GKKEHQFYLWFDPTAD.FHTYKIVWNP
TaXTH3    IDIEFMGNLSG..DPYVMNTNVWAGGD......GKKEHQFYLWFDPTAD.FHTYKIVWNP
TaXTH4    IDIEFMGNSSG..DPYVMNTNVWASGD......GKKEHQFYLWFDPSAD.FHTYKITWNP
TaXTH5    IDFEFLGNVTG..EPYTLHTNVFAKGQ......GQREQQFRLWFDPTKA.FHTYSIIWNP
TaXTH6.1a LDFELLGNRRG..HGWRVQTNMYGNGS....TARGREERYHLPVEPTVAGVHRYAIAWTP
TaXTH6.1d LDFELLGNRRG..HGWRVQTNMYGNGS....TARGREERYHLPVEPTVAGVHRYAIAWTP
TaXTH6.2a IDIEFMGNLSG..DPYVMNTNVWASGD......GKKEHQFYLWFDPTAD.FHTYKIVWNP
TaXTH6.2d IDIEFMGNLSG..DPYVMNTNVWASGD......GKKEHQFYLWFDPTAD.FHTYKIVWNP
TaXTH7.1a VDLEFLGNSSG..EPYTLHTNIYARGR......GGREKQYRLWFDPTQD.FHTYSILWNP
TaXTH7.1b VDLEFLGNSSG..EPYTLHTNIYARGR......GGREKQYRLWFDPTQD.FHTYCILWNP
TaXTH7.1d VDLEFLGNSSG..EPYTLHTNIYARGR......GGREKQYRLWFDPTQD.FHTYSILWNP
TaXTH7.2a VDLEFLGNSSG..EPYTLHTNIYARGR......GGREKQYRLWFDPTQD.FHTYSILWNP
TaXTH7.2b IDFEFLGNETG..QPYTLHTNVYAAGV......GGKEMQFRPWFDPTDG.YHNYTISWTP
TaXTH7.2d LDFEFLGNRTG..EPYIIQTNVYRSGV......GGREMRHSLWFDPTAE.FHSYSILWNP
TaXTH7.3a IDFEFLGNVSG..EPYLMQTNVFAQGV......GGREQRSYLWFDPTED.FHNYTILWNP
TaXTH7.3b IDFEFLGNVSG..EPYLMQTNVFAQGV......GGREQRSYLWFDPTED.FHNYTILWNP
TaXTH7.3d IDFEFLGNVSG..EPYLMQTNVFTQGV......GGREQRSYLWFDPTED.FHNYTILWNP
TaXTH7.4a IDLEFLGNSTG..EPYTLHTNVYINGT......GSKEQQFHLWFDPAAD.FHTYSIVWTP
TaXTH7.4b IDLEFLGNSTG..EPYTLHTNVYINGT......GSKEQQFHLWFDPTAD.FHTYSIVWTP
TaXTH7.4d IDLEFLGNSTG..EPYTLHTNVYINGT......GSKEQQFHLWFDPTAD.FHTYSIVWTP
TaXTH7.5a IDLEFLGNATG..QPYTLHTNIFANGE......GGREVQYRLWFDPTKD.FHTYSIVWNT
TaXTH7.5b IDLEFLGNATG..QPYTLHTNIFANGE......GGREVQYRLWFDPTQD.FHTYSIVWNT
TaXTH7.5d IDLEFLGNATG..QPYTLHTNIFANGE......GGREVQYRLWFDPTQD.FHTYSIVWNT
TaXTH7.6a IDFEFLGNETG..QPYTLHTNVFADGV......GKKEMQFVPWFDPTAD.FHAYTISWTP
TaXTH7.6d IDFEFLGNQTG..QPYTLHTNVFADGV......GKKEMQFVPWFDPTAD.FHAYTISWTP
TaXTH7.7d IDFEFLGNETG..QPYTLHTNVFADGV......GKKEMQFVPWFDPTAD.FHAYTISWTP
TaXTH8.1a VDFEFLGNVDG..KPVALQTNIFLNGQ......GYREQKFYLWFDPSAA.VHDYKILWNQ
TaXTH8.1b IDMEFMGNSTG..HPVVLNTNVWANGD......GKKEHQFDLWFDPAAA.YHTYTIIWNP
TaXTH8.2b IDMEFMGNSSGPGHPVVLNTNVWVNGD......GKKEHQFNLWFDPAAD.FHTYTIIWNP
TaXTH8.3b VDFEFLGNVDG..KPVALQTNIFLNGQ......GYREQKFYLWFDPSAA.VHDYKILWNQ
TaXTH8.4b VDFEFLGNVDG..KPVALQTNIFLNGQ......GYREQKFYLWFDPAAA.VHDYKILWNQ
TaXTH9.1a LDFEFLGNVKG..KEWRVQTNVYGDGS....TAVGREERYGLWFDPTQD.FHRYAILWTN
TaXTH9.1b LDFEFLGNVKG..KEWRVQTNVYGDGS....TAVGREERYGLWFDPTQD.FHRYAILWTN
TaXTH9.1d IDFEFLGNRTG..QPYILQTNVFSGGK......GDREQRIYLWF..................
TaXTH9.2a IDFEFLGNRTG..QPYILQTNVFSGGK......GDREQRIYLWFDPTKD.YHSYSVLWNL
TaXTH9.2b IDVELLGTVPG..EPYTLQTNVYVRGTGDAHPIVGREMRFHLWFDPAAA.FHHYAVLWNP
TaXTH9.2d LDFEFLGNVKG..KEWRVQTNVYGDGS....TAVGREERYGLWFDPTQD.FHRYAILWTN
TaXTH9.3a LDFEFLGNRAG..HRWRMQTNVYGNGS....TARGREERYMLPFDPTAA.KHRFSILWSS
TaXTH9.3d IDVELLGTVPG..EPYTLQTNVYVRGTGDAHPIVGREMRFHLWFDPAAA.FHHYAVLWNP
TaXTH9.4a IDMEFMGNSTG..QPVALNTNVWANGD......GKKEQQFYLWFDPAAD.YHTYTIIWND
TaXTH10.1a IDVELLGTVPG..EPYTLQTNVYVRGTGDAHPIVGREMRFHLWFDPAAA.FHHYAVLWNP
TaXTH10.1b IDMEFMGNSTG..QPVVLNTNVWANGD......GKKEHQFDLWFDPAAD.YHTYTIIWNH
TaXTH10.1d IDMEFMGNSTG..QPVVLNTNVWANGD......GKKEHQFDLWFDPAAD.YHTYTIIWND
TaXTH11.1a LDFEFLGNIRG..KPWRMQTNMYGNGS....VSRGREERYVLPFDPTTE.FHRYSILWAR
TaXTH11.1b LDFEFLGNIRG..KPWRMQTNMYGNGS....VSRGREERYVLPFDPTTE.FHRYSILWAR
TaXTH11.1d VDLEFLGNVTG..EPYTLHTNIFANGV......GNREEQFRLWFDPAAD.FHTYSIDWNP
TaXTH11.2a LDFEFLGSRWG..GQWRVQTNVYGNGS....TSRGREERYLLPFDPTLE.AHRYSILWAP
TaXTH11.2b LDFEFLGSRWG..GQWRVQTNVYGNGS....TSRGREERYLLPFDPTLE.AHRYSILWAP
TaXTH11.3a VDLEFLGNVTG..EPYTLHTNIFANGV......GNREEQFRLWFDPAAG.FHTYSIDWNP
TaXTH11.3b VDLEFLGNVTG..EPYTLHTNIFANGV......GNREEQFRLWFDPTAD.FHTYSIDWNP
TaXTH11.4b IDIEFMGNSSG..DPYVMNTNVWASGD......GKKEHQFYLWFDPSAD.FHTYKITWNP
TaXTH12.1a IDMEFMGNATG..QPVVLNTNVWANGD......GKKEHQFDLWFDPAAD.FHTYTIIWND
TaXTH12.1b VDFEFLGNVDG..ENITLQTNVFVNGD......GDREQRLNLWFDPAAD.FHEYKILWNP
TaXTH12.1d IDMEFMGNATG..QPVVLNTNVWANGD......GKKEHQFDLWFDPAAD.FHTYTIIWNH
TaXTH12.2a IDMEFMGNTTG..QPVALNTNVWANGD......GKKEQQFYLWFDPAAD.YHTYTIIWNA
TaXTH12.2b IDFEFLGNETG..QPYTIHTNVFADGV......GAKEVQFYPWFDPTDD.FHNYTILWNP
TaXTH12.2d IDMEFMGNSTG..QPVALNTNVWANGD......GKKEQQFYLWFDPAAD.YHTYTIIWNA
TaXTH12.3a VDFEFLGNVDG..ENITLQTNVFVNGD......GDREQRLNLWFDPAAD.FHEYKILWNP
TaXTH12.3b IDMEFLGTIPG..EPYTLQTNVYVRGSGDGR.IIGREMRFHLWFDPTAG.FHNYAILWNP
TaXTH12.3d VDFEFLGNVDG..ENITLQTNVFVNGD......GDREQRLNLWFDPAAD.FHEYKILWNP
TaXTH12.4a IDFEFLGNETG..QPYTIHTNVFADGV......GAKEVQFYPWFDPTAD.FHNYTIFWNP
TaXTH12.4b IDFEFLGNVTG..EPYTLHTNVFTQGQ......GQREQQFRLWFDPTND.FHTYSILWNP
TaXTH12.4d IDFEFLGNETG..QPYTIHTNVFADGV......GAKEVQFYPWFDPTDD.FHNYTILWNP
TaXTH12.5a IDFEFLGNVTG..EPYTLHTNVFTQGQ......GQREQQFRLWFDPTND.FHTYSILWNP
TaXTH12.5d IDMEFLGTIPG..EPYTLQTNVYVRGSGDGR.IIGREMRFHLWFDPAAG.FHNYAILWNP
TaXTH12.6a IDFEFLGNVTG..EPYTLHTNVFTQGQ......GQREQQFRLWFDPTND.FHTYSILWNP
TaXTH12.6d IDFEFLGNVTG..EPYTLHTNVFTQGQ......GQREQQFRLWFDPTND.FHTYSILWNP
TaXTH12.7a IDFEFLGNASG..QPYTVHTNVYSQGK......GGREQQFRMWFDPTAD.FHTYSVVWNP
TaXTH12.7d IDFEFLGNVTG..EPYTLHTNVFTQGQ......GQREQQFRLWFDATND.FHTYSILWNP
TaXTH12.8d IDFEFLGNVTG..EPYTLHTNVFTQGQ......GQREQQFRLWFDPTND.FHTYSILWNP
```

```
              β11      β12       η3                           β13
1un1         ------->TT------>   ααα                         -------->
             160       170                 180       190
1un1         YMIVFLVDDVPIRVFKN.CKDL................GVKFPFNQPMKIYSSLWN..AD
TaXTH1       KNIIFQVDDVPVRTFKK.YDD..................LPYPSSQPMTVHATLWD..GS
TaXTH2       KNIIFQVDDVPVRTFKK.YDD..................LPYPSSQPMTVHATLWD..GS
TaXTH3       KNIIFQVDDVPVRTFKK.YDD..................LPYPSSQPMMVHATLWD..GS
TaXTH4       KNIIFEVDGVPVRTFKK.YDG..................LPFPSSRPMTVHATLWD..GS
TaXTH5       QHVIFAVDGTAIRDFKN.HEAR................GVSFPKSQPMRLYASLWN..AD
TaXTH6.1a    NNIVFYLDGVPIREVVRVPS.................MGGDFPS.KPMSVYATIWD..GS
TaXTH6.1d    NNIVFYLDGVPIREVVGVPS.................MGGDFPS.KPMSVYATIWD..GS
TaXTH6.2a    KNIIFQVDDVPVRTFKK.YDD..................LPYPSSQPMTVHATLWD..GS
TaXTH6.2d    KNIIFQVDDVPVRTFKK.YDD..................LPYPSSQPMTVHATLWD..GS
TaXTH7.1a    KEILIIVDGTPVRQMKN.QQRK................DIPFPLYQPMRLYSSIWN..AE
TaXTH7.1b    KEILILVDGIPIRQMKN.QQRK................DIPFPLYQPMRLYSSIWN..AE
TaXTH7.1d    KEILILVDGTPVRQMKN.QQRK................DIPFPLYQPMRLYSSIWN..AE
TaXTH7.2a    KEILIIVDGTPVRQMKN.QQRK................DIPFPLYQPMRLYSSIWN..AE
TaXTH7.2b    CTIVWYVDGTPIRAFRNYERTH................GVAFPTSRPMYAYSSIWA..AE
TaXTH7.2d    KQIVFFVDKVPIREYRN.SDKP................NRFFPIMKPMYVFSSIWN..AD
TaXTH7.3a    LNIIFSVDGVPVRVFRN.HDAN................GVPYLSRQAMKVHATIWD..GD
TaXTH7.3b    LNIIFSVDGVPVRVFRN.HDAN................GVPYLTRQAMKVHATIWD..GD
TaXTH7.3d    LNIIFSVDGVPVRVFRN.HDAN................GVPYLSRQAMKVHATIWD..GD
TaXTH7.4a    LHILVLVDGTPIRELRN.HADK................GVAYPSWQRMRLYGSLWN..AE
TaXTH7.4b    LHLLVLVDGTPIRELKN.HADR................GVAYPSWQRMRLYGSLWN..AE
TaXTH7.4d    LHLLVLVDGTPIRELKN.HADR................GVAYPSWQRMRLYGSLWN..AE
TaXTH7.5a    DEILILVDGVPIRQFKN.HWDA................GVPFPVYQPMRLFGCLWD..AD
TaXTH7.5b    DEILILVDGVPIRQFKN.HWDA................GVPFPVYQPMRLFGCLWN..AD
TaXTH7.5d    DEILILVDGVPIRQFKN.HWDA................GVPFPVYQPMRLFGCLWD..AD
TaXTH7.6a    CMIVWYVDDVPIRVFRNYR.DK................GIAYPIKRPMFGYSSIWS..AE
TaXTH7.6d    CMIVWYVDDVPIRVFRNYR.DK................GIAYPIKRPMFGYSSIWS..AE
TaXTH7.7d    CMIVWYVDDVPIRVFRNYR.DK................GIAYPIKRPMFGYSSIWS..AE
TaXTH8.1a    HQLVMLVDETPIRVLKNLPGRT...............PGYQFPS.RPMKIRASIWD..GS
TaXTH8.1b    ENILFKVDNLFIRSFKR.FAG..................LPYPTSKPMRLHATLWD..GS
TaXTH8.2b    ENILFKVDNLFIRSFKR.FAG..................IPYTSSKPMRLHATLWD..GS
TaXTH8.3b    HQLVMLVDEMPIRVLKNLPGRK...............PGYQFPS.RPMKIRASIWD..GS
TaXTH8.4b    HQLVMLVDETPIRVLKNLAGRT...............PAYQFLT.RPMRIRASIWD..GS
TaXTH9.1a    RTIVFYVDGTPIREVVRSEA.................MGAQFPS.KPMSLYATIWD..GS
TaXTH9.1b    RTIVFYVDGTPIREVVRSEA.................MGAQFPS.KPMSLYATIWD..GS
TaXTH9.1d    .....FVDDTPIRVFKN.SKDL................GVRYPFDQPMKLYSSLWN..AD
TaXTH9.2a    YMIAFFVDDTPIRVFKN.SKDL................GVRYPFDQPMKLYSSLWN..AD
TaXTH9.2b    DEIVFLVDDVPVRRYQKKVE...................ATFPE.REMWAYGSVWD..AS
TaXTH9.2d    RTIVFYVDGTPIREVVRSEA.................MGAQFPS.KPMSLYATIWD..GS
TaXTH9.3a    SSVVFYVDGTPVREAQRRRSRRRFTDDGMGPDGDDSDMGADYPS.KPMAVYVTIWD..GS
TaXTH9.3d    DEIVFLVDDVPVRRYQKKVE...................ATFPE.REMWAYGSVWD..AS
TaXTH9.4a    RNIIFKVDDLVLRCFTR.HAD..................LPYPGAKPMSVHATLWD..GS
TaXTH10.1a   DEIVFLVDDVPVRRYQKKVE...................ATFPE.REMWAYGSVWD..AS
TaXTH10.1b   KNVLFKVDDLFIRSFTR.YAD..................LPYPGVKPMSVHATLWD..GS
TaXTH10.1d   KNVLFKVDDLFIRSFRR.HAD..................LPYPGAKPMSVHATLWD..GS
TaXTH11.1a   DAVVFYVDDVPVRH.LRHAR.................AGRDFPA.KPMSLYATVWD..AS
TaXTH11.1b   DAVVFYVDDVPVRN.LRRAR.................AGRDFPA.KPMSLYATVWD..AS
TaXTH11.1d   KRITILVDGVPIRSFRN.KEEH................GVAFPTWQKMRLHGSLWN..AD
TaXTH11.2a   THIIFYVDDTPIREVVRHPG.................MGGDFPA.KPMAVYATIWD..GS
TaXTH11.2b   THIIFYVDDTPIREVVRHPG.................MGGDFPA.KPMAVYATIWD..GS
TaXTH11.3a   KRITILVDGVPIRSFRN.KEEH................GVPFPTWQKMRLHGSLWN..AD
TaXTH11.3b   KRITILVDGVPIRSFRN.KEEH................GVAFPTWQKMRLHGSLWN..AD
TaXTH11.4b   KNIIFEVDGVPVRTFKK.YDG..................LPFPSSRPMTVHATLWD..GS
TaXTH12.1a   KNVLFKVDDLFIRSFRR.YAD..................LPYPGAKPMSVHATLWD..GS
TaXTH12.1b   YQLVILVDDVPIRVLRNLTGQV...............PEYEFPA.KQMGVRASLWD..GS
TaXTH12.1d   KNVLFKVDNLFIRSFKR.YAD..................LPYPGAKPMSVHATLWDWDGS
TaXTH12.2a   KNVIFKVDGLFVRCFTR.HAD..................LPYPGAKPMAVHATLWD..GS
TaXTH12.2b   SMIVWFVDSIPIRVFRNYA.SK................GVPFPTKRPMYGFSSIWS..AD
TaXTH12.2d   KNVIFKVDDLFLRCFTR.HAD..................LPYPGAKPMAVHATLWD..GS
TaXTH12.3a   YQLVILVDDVPIRVLRNLTGQV...............PEYEFPA.KQMGVRASLWD..GS
TaXTH12.3b   DAITFFVDDVPIRRYERKTE...................LTFPD.RPMWAYGSIWD..AS
TaXTH12.3d   YQLVILVDDVPIRVLRNLTGQV...............PEYEFPV.KQMGVRASLWD..GS
TaXTH12.4a   SMIVWFVDSIPIRVFRNYA.SK................GVPFPTKRPMYGFSSIWS..AD
TaXTH12.4b   KHIIFMVDDMPIRDFKN.LEGK................GIAFPKNQPMRLYSSLWN..AD
TaXTH12.4d   SMIVWFVDSIPIRVFRNYA.SK................GVPFPTKRPMYGFSSIWS..AD
TaXTH12.5a   KHIIFMVDDMPIRDFRN.LEGK................GIAFPKNQPMRLYSSLWN..AD
TaXTH12.5d   DAITFFVDDVPIRRYERKTE...................LTFPD.RPMWAYGSIWD..AS
TaXTH12.6a   KHIIFMVDDMPIRDFRN.LEGK................GIAFPKNQPMRLYSSLWN..AD
TaXTH12.6d   KHIIFMVDDMPIRDFRN.LEGK................GIAFPKNQPMRLYSSLWN..AD
TaXTH12.7a   THILFYVDGTPIREHRNREAAT................GVAYPRSQAMRVYASVWD..AE
TaXTH12.7d   KHIIFMVDDMPIRDFKN.LEGK................GIAFPKNQPMRLYSSLWN..AD
TaXTH12.8d   KHIIFMVDDMPIRDFRN.LEGK................GIAFPKNQPMRLYSSLWN..AD
```

```
                        η4       η5        β14                                   η6
1un1                                       ───────────►
            200        210       220       230                 240
1un1        DWATRGGLEKTDWSKAPFIASYRSFHIDGCEAS...........VEAKFCATQGARWWD
TaXTH1      YWATRHGDVKIDWSQAPFVVNYRGYSSNGCVSN.........GGS..SACPAGSDAWMN
TaXTH2      YWATRHGDVKIDWTQAPFVVNYRGYSSNGCVSN.........GGS..SACPAGSDAWMN
TaXTH3      YWATRHGDVKIDWSQAPFVVNYRGYSSNGCVSS.........GGS..SACPAGSDAWMN
TaXTH4      YWATQHGTVKIHWRHHPFVVPYKAYHANGCVHD.........KATNKTACPAGSDAWMR
TaXTH5      DWATQGGRVKTDWSKAPFVASFRNFNADACVMSG.GAQRC..PAGTMEASAAGGSGSWWN
TaXTH6.1a   AWATDGGKYKVDYAYAPFAAEFSDLVLSGCDASSVADPEG.............CQVDLL
TaXTH6.1d   AWATDGGKYKVDYAYAPFAAEFSDLVLSGCDG..................CQVDLL
TaXTH6.2a   YWATRHGDVKIDWTQAPFVVNYRGYSSNGCVSN.........GGS..SACPAGSDAWMN
TaXTH6.2d   YWATRHGDVKIDWSQAPFVVNYRGYSSNGCVSN.........GGS..SACPAGSDAWMN
TaXTH7.1a   DWATQGGRVKTDWSQAPFTSLFRNYSAVSCV.....SQKT.AWICGR...GSSDSS.WFT
TaXTH7.1b   DWATQGGRVKTDWSQAPFTSLFRNYSVVACV.....PQKT.AWICGR...GSSDSS.WFT
TaXTH7.1d   DWATQGGRVKTDWSQAPFTSLFRNYSAVSCV.....SQKT.AWICGR...GSSDSS.WFT
TaXTH7.2a   DWATQGGRVKTDWSQAPFTSLFRNYSAVSCV.....SQKT.AWICGR...GSSDSS.WFT
TaXTH7.2b   DWATQGGRVRADWTRAPFVASYRGIDLDVCECY........GGD..CVYTCAAAFG...
TaXTH7.2d   DWATRGGLEKTDWTKGPFVSSYSDFTADACAWAT........GPAPPACAAATGNSWWD
TaXTH7.3a   TWATRGGRVKIDWAHAPFVASYGTYASSACVSAA...GNGDQDGAPSAFCCPGDASSWMA
TaXTH7.3b   TWATRGGRVKIDWAHAPFVASYGTYAASACVSAA...GNGDQDGAPSAFCCPGDAPSWMA
TaXTH7.3d   TWATRGGRVKIDWAHAPFVASYGTYAASACVSAA...GNDDQEGAPSAFCCPGDASSWMA
TaXTH7.4a   DWATQGGRVKTDWSLAPFVAQYRNFTAT..................TSSPGAGGGYYD
TaXTH7.4b   DWATQGGRVKTDWSLAPFVAQYRNFTAT..................TSSPGAGGGYYD
TaXTH7.4d   DWATQGGRVKTDWSLAPFVAQYRNFTAT..................TSSPGAGGGYYD
TaXTH7.5a   DWATQGGRVKTDWSQAPFVAYFRNYTASGCA.....PSAGGSWACGPDPSGSGGSSGWMD
TaXTH7.5b   DWATQGGRVKTDWSQAPFIAYFRNYTASGCA.....PTAGGSWACDQNPSGSGSSSGWMD
TaXTH7.5d   DWATQGGLVKTDWSQAPFVAYFKNYTASGCA.....PSAGGSWVCGQDPSGSGGSPGWMD
TaXTH7.6a   DWATQGGRVKADWSKAPFVAGYRDMVLDVCPCD........GADS.CVYGCAGAFSHGG
TaXTH7.6d   DWATQGGRVKADWSKAPFVAGYRDMVLDVCPCD........GADS.CVYGCAGAFSHGG
TaXTH7.7d   DWATQGGRVKADWSKAPFVAGYRDMVLDVCPCD........GADS.CVYGCAGAFSHGG
TaXTH8.1a   SWATDNGNIRVDWNRAPFTSAFQRFNVDACPATRGAPCGS.............PNLWWN
TaXTH8.1b   YWATEKGKIPINWSNAPFVVSYRNFYANACVSG.............GACHAGSGRWMK
TaXTH8.2b   FWATEKGKVPIDWSNAPFNVLYRNYYANACVSG.............GACHAGSDGWMN
TaXTH8.3b   SWATDNGKIRVDWNRAPFTSAFQRFNVDACPATGGAPCGS.............PNLWWN
TaXTH8.4b   PWATDNGKIKVDWNRAPFTSAFQRFNVDACPATGGAPCGS.............PNLWWN
TaXTH9.1a   SWATSGGRYKVDYKYAPYVAEFTDLELHGCAHD.................QAQSACE
TaXTH9.1b   SWATSGGRYKVDYKYAPYVAEFTDLELRGCD....................QPAACE
TaXTH9.1d   DWATRGGREKTDWSKAPFVASYRGFHVDGCEAS...........AEAKFCATQGARWWD
TaXTH9.2a   DWATRGGREKTDWSKAPFVASYRGFHVDGCEAS...........AEAKFCATQGARWWD
TaXTH9.2b   DWATDGGRYRSDYRYQPFVSGFKDFKVAGCEVGAPAS................CGPVPA
TaXTH9.2d   SWATSGGRYKVDYKYAPYVAEFTDLELRGCAH..................APPASCE
TaXTH9.3a   TWATENGKHTVDYGHGPFTAEFSGLVLRGCPADAGADIRQLHLG..ASTQRCATAEWELM
TaXTH9.3d   DWATDGGRYRSDYRYQPFVSGFKDFKVAGCEVGAPAS................CGPVPA
TaXTH9.4a   YWATQRGKVKVDWSAAPFVVSYRGYLADACAPA.........GAGRPLACPAGTGRWMR
TaXTH10.1a  DWATDGGRYRSDYRYQPFVSGFKDFKVAGCEVGAPAS................CSPVPA
TaXTH10.1b  YWATLKGKVPVDWSGAPFLVSYRAYSADACVPA........ADAG.PLSCPAGTDRWMN
TaXTH10.1d  YWATQKGKVPVDWSGAPFVVSYRAYSADACVPA........EGS..PLSCPAGTDRWMN
TaXTH11.1a  NWATSGGRYRVDYHYGPFVASFTDLALAGCRASSPTEEG..............CADALA
TaXTH11.1b  NWATSGGRYRVNYQYGPFVASFTDLALAGCRAPSPTEEG..............CADALA
TaXTH11.1d  DWATQGGRVKTDWSEAPFFAHYRNLRVSWCR.....PSPGVAWCGDE....PPGST.WFD
TaXTH11.2a  AWATEGGKYKVNYKYAPFASDFSDLSLLGCRAAAAADPAALRLGRSSDAAAAGCDLLGLL
TaXTH11.2b  AWATEGGKYKVNYKYAPFASDFSDLSLRGCR...VADPAALRLR..SDAAG.GYDLLGLM
TaXTH11.3a  DWATQGGRVKTDWSEAPFFAHYRNLRVSWCR.....PPPGVAWCGDE....PPGST.WFD
TaXTH11.3b  DWATQGGRVKTDWSEAPFFAHYRNLRVSWCR.....PSPGLAWCGDE....PPEST.WFD
TaXTH11.4b  YWATQHGTVKIHWRHAPFVVPYKAYHANGCVHD.........KATNKTSCPAGSDAWMR
TaXTH12.1a  YWATQKGKVPVDWSGAPFVVSYRGYTADACVPN........GGEGSPLSCPAGTDRWMN
TaXTH12.1b  DWATDGGRIKIDWGRAPFAAGFQGFDVDACANTSSTPCDS.............TDLWWN
TaXTH12.1d  YWATQKGKVPVDWSGAPFVVSYRAYSVDACVPAD.......GGEGSLLSCPAGTDRWMN
TaXTH12.2a  YWATQRGKVKVDWAAAPFVASYRGYSADACVPA.........GAGRPLACPADTGRWMR
TaXTH12.2b  DWATQGGRVKTDWTKAPFVAEYNNMGLDVCECS........GADAECAARCNKDTP...
TaXTH12.2d  YWATQRGKVKVDWTTAPFVASYRGYSADACVPA.........GAGRPLACPAGTGRWMR
TaXTH12.3a  DWATDGGRIKIDWGRAPFTAGFRGFDVDACANTSSTPCDS.............TDLWWN
TaXTH12.3b  DWATDHGRYRADYRYQPFVARFDRFVVAGCGPSAPPS................CRPARA
TaXTH12.3d  DWATDGGRIKIDWGRAPFTAGFQGFDVDACANTSSTPCDS.............TDLWWN
TaXTH12.4a  DWATQGGRVKTDWAKAPFVAEYDNMGLDVCECL........STDAECAARCNKDTP...
TaXTH12.4b  DWATQGGRVKTDWSHAPFSASYRGFKADACVVTAGGRPRCGSSVGTEVAPGTGAAGEWYN
TaXTH12.4d  DWATQGGRVKTDWTKAPFVAEYDNMGLHVCECS........STDDECATRCNKDTP...
TaXTH12.5a  DWATQGGRVKTDWSHAPFSASYRGFKADACVVTAGGKPHCGASVGTEVAPGTGAAGEWYN
TaXTH12.5d  DWATDHGRYRADYRYQPFVARFDRFVVAGCGPGAPPS................CRPARA
TaXTH12.6a  DWATQGGRVKTDWSHAPFSASYRGFKADACVVTAGGRPRCGASVGTEVAPGTGATGEWYN
TaXTH12.6d  DWATQGGRVKTDWSHAPFSASYRGFKADACVVTAAGRPRCGASVGTEVAPGTGAAGEWYN
TaXTH12.7a  EWATQGGRVRTDWSRAPFVASYKGFAASGC............ASQDAAACARSNGAWMY
TaXTH12.7d  DWATQGGRVKTDWSHAPFSASYRGFKADACVVTAAGRPRCGSSVGTEVAPGTGAAGEWYN
TaXTH12.8d  DWATQGGRVKTDWSHAPFSASYRGFKADACVVTAAGRPHCGASVGTDVAPGTGAAGEWYN
                                          1                         1
```

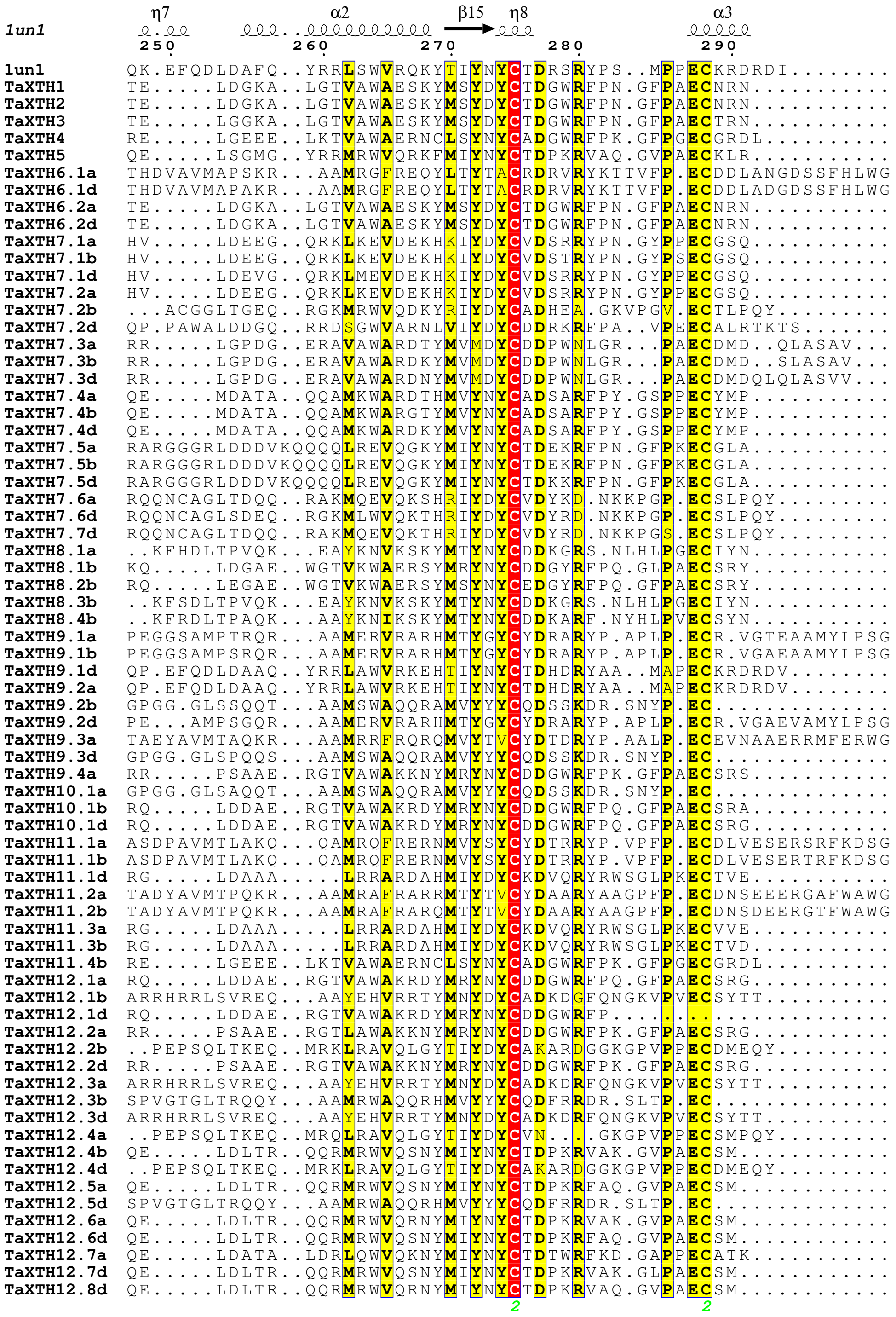

η7 α2 β15 η8 α3
1un1
250 260 270 280 290
1un1 QK.EFQDLDAFQ..YRRLSWVRQKYTIYNYCTDRSRYPS..MPPECKRDRDI........
TaXTH1 TE.....LDGKA..LGTVAWAESKYMSYDYCTDGWRFPN.GFPAECNRN...........
TaXTH2 TE.....LDGKA..LGTVAWAESKYMSYDYCTDGWRFPN.GFPAECNRN...........
TaXTH3 TE.....LGGKA..LGTVAWAESKYMSYDYCTDGWRFPN.GFPAECTRN...........
TaXTH4 RE.....LGEEE..LKTVAWAERNCLSYNYCADGWRFPK.GFPGECGRDL..........
TaXTH5 QE.....LSGMG..YRRMRWVQRKFMIYNYCTDPKRVAQ.GVPAECKLR...........
TaXTH6.1a THDVAVMAPSKR...AAMRGFREQYLTYTACRDRVRYKTTVFP.ECDDLANGDSSFHLWG
TaXTH6.1d THDVAVMAPAKR...AAMRGFREQYLTYTACRDRVRYKTTVFP.ECDDLADGDSSFHLWG
TaXTH6.2a TE.....LDGKA..LGTVAWAESKYMSYDYCTDGWRFPN.GFPAECNRN...........
TaXTH6.2d TE.....LDGKA..LGTVAWAESKYMSYDYCTDGWRFPN.GFPAECNRN...........
TaXTH7.1a HV.....LDEEG..QRKLKEVDEKHKIYDYCVDSRRYPN.GYPPECGSQ...........
TaXTH7.1b HV.....LDEEG..QRKLKEVDEKHKIYDYCVDSTRYPN.GYPSECGSQ...........
TaXTH7.1d HV.....LDEVG..QRKLMEVDEKHKIYDYCVDSRRYPN.GYPPECGSQ...........
TaXTH7.2a HV.....LDEEG..QRKLKEVDEKHKIYDYCVDSRRYPN.GYPPECGSQ...........
TaXTH7.2b ...ACGGLTGEQ..RGKMRWVQDKYRIYDYCADHEA.GKVPGV.ECTLPQY.........
TaXTH7.2d QP.PAWALDDGQ..RRDSGWVARNLVIYDYCDDRKRFPA..VPEECALRTKTS.......
TaXTH7.3a RR.....LGPDG..ERAVAWARDTYMVMDYCDDPWNLGR...PAECDMD..QLASAV...
TaXTH7.3b RR.....LGPDG..ERAVAWARDKYMVMDYCDDPWNLGR...PAECDMD..SLASAV...
TaXTH7.3d RR.....LGPDG..ERAVAWARDNYMVMDYCDDPWNLGR...PAECDMDQLQLASVV...
TaXTH7.4a QE.....MDATA..QQAMKWARDTHMVYNYCADSARFPY.GSPPECYMP...........
TaXTH7.4b QE.....MDATA..QQAMKWARGTYMVYNYCADSARFPY.GSPPECYMP...........
TaXTH7.4d QE.....MDATA..QQAMKWARDKYMVYNYCADSARFPY.GSPPECYMP...........
TaXTH7.5a RARGGGRLDDDVKQQQQLREVQGKYMIYNYCTDEKRFPN.GFPKECGLA...........
TaXTH7.5b RARGGGRLDDDVKQQQQLREVQGKYMIYNYCTDEKRFPN.GFPKECGLA...........
TaXTH7.5d RARGGGRLDDDVKQQQQLREVQGKYMIYNYCTDKKRFPN.GFPKECGLA...........
TaXTH7.6a RQQNCAGLTDQQ..RAKMQEVQKSHRIYDYCVDYKD.NKKPGP.ECSLPQY.........
TaXTH7.6d RQQNCAGLSDEQ..RGKMLWVQKTHRIYDYCVDYRD.NKKPGP.ECSLPQY.........
TaXTH7.7d RQQNCAGLTDQQ..RAKMQEVQKTHRIYDYCVDYRD.NKKPGS.ECSLPQY.........
TaXTH8.1a ..KFHDLTPVQK...EAYKNVKSKYMTYNYCDDKGRS.NLHLPGECIYN...........
TaXTH8.1b KQ.....LDGAE..WGTVKWAERSYMRYNYCDDGYRFPQ.GLPAECSRY...........
TaXTH8.2b RQ.....LEGAE..WGTVKWAERSYMSYNYCEDGYRFPQ.GFPAECSRY...........
TaXTH8.3b ..KFSDLTPVQK...EAYKNVKSKYMTYNYCDDKGRS.NLHLPGECIYN...........
TaXTH8.4b ..KFRDLTPAQK...AAYKNIKSKYMTYNYCDDKARF.NYHLPVECSYN...........
TaXTH9.1a PEGGSAMPTRQR...AAMERVRARHMTYGYCYDRARYP.APLP.ECR.VGTEAAMYLPSG
TaXTH9.1b PEGGSAMPSRQR...AAMERVRARHMTYGYCYDRARYP.APLP.ECR.VGAEAAMYLPSG
TaXTH9.1d QP.EFQDLDAAQ..YRRLAWVRKEHTIYNYCTDHDRYAA..MAPECKRDRDV........
TaXTH9.2a QP.EFQDLDAAQ..YRRLAWVRKEHTIYNYCTDHDRYAA..MAPECKRDRDV........
TaXTH9.2b GPGG.GLSSQQT...AAMSWAQQRAMVYYYCQDSSKDR.SNYP.EC..............
TaXTH9.2d PE...AMPSGQR...AAMERVRARHMTYGYCYDRARYP.APLP.ECR.VGAEVAMYLPSG
TaXTH9.3a TAEYAVMTAQKR...AAMRRFRQRQMVYTVCYDTDRYP.AALP.ECEVNAAERRMFERWG
TaXTH9.3d GPGG.GLSPQQS...AAMSWAQQRAMVYYYCQDSSKDR.SNYP.EC..............
TaXTH9.4a RR.....PSAAE..RGTVAWAKKNYMRYNYCDDGWRFPK.GFPAECSRS...........
TaXTH10.1a GPGG.GLSAQQT...AAMSWAQQRAMVYYYCQDSSKDR.SNYP.EC..............
TaXTH10.1b RQ.....LDDAE..RGTVAWAKRDYMRYNYCDDGWRFPQ.GFPAECSRA...........
TaXTH10.1d RQ.....LDDAE..RGTVAWAKRDYMRYNYCDDGWRFPQ.GFPAECSRG...........
TaXTH11.1a ASDPAVMTLAKQ...QAMRQFRERNMVYSYCYDTRRYP.VPFP.ECDLVESERSRFKDSG
TaXTH11.1b ASDPAVMTLAKQ...QAMRQFRERNMVYSYCYDTRRYP.VPFP.ECDLVESERTRFKDSG
TaXTH11.1d RG.....LDAAA.....LRRARDAHMIYDYCKDVQRYRWSGLPKECTVE...........
TaXTH11.2a TADYAVMTPQKR...AAMRAFRARRMTYTVCYDAARYAAGPFP.ECDNSEEERGAFWAWG
TaXTH11.2b TADYAVMTPQKR...AAMRAFRARQMTYTVCYDAARYAAGPFP.ECDNSDEERGTFWAWG
TaXTH11.3a RG.....LDAAA.....LRRARDAHMIYDYCKDVQRYRWSGLPKECVVE...........
TaXTH11.3b RG.....LDAAA.....LRRARDAHMIYDYCKDVQRYRWSGLPKECTVD...........
TaXTH11.4b RE.....LGEEE..LKTVAWAERNCLSYNYCADGWRFPK.GFPGECGRDL..........
TaXTH12.1a RQ.....LDDAE..RGTVAWAKRDYMRYNYCDDGWRFPQ.GFPAECSRG...........
TaXTH12.1b ARRHRRLSVREQ...AAYEHVRRTYMNYDYCADKDGFQNGKVPVECSYTT..........
TaXTH12.1d RQ.....LDDAE..RGTVAWAKRDYMRYNYCDDGWRFP......................
TaXTH12.2a RR.....PSAAE..RGTLAWAKKNYMRYNYCDDGWRFPK.GFPAECSRG...........
TaXTH12.2b ..PEPSQLTKEQ..MRKLRAVQLGYTIYDYCAKARDGGKGPVPPECDMEQY.........
TaXTH12.2d RR.....PSAAE..RGTVAWAKKNYMRYNYCDDGWRFPK.GFPAECSRG...........
TaXTH12.3a ARRHRRLSVREQ...AAYEHVRRTYMNYDYCADKDRFQNGKVPVECSYTT..........
TaXTH12.3b SPVGTGLTRQQY...AAMRWAQQRHMVYYYCQDFRRDR.SLTP.EC..............
TaXTH12.3d ARRHRRLSVREQ...AAYEHVRRTYMNYDYCADKDRFQNGKVPVECSYTT..........
TaXTH12.4a ..PEPSQLTKEQ..MRQLRAVQLGYTIYDYCVN.....GKGPVPPECSMPQY.........
TaXTH12.4b QE.....LDLTR..QQRMRWVQSNYMIYNYCTDPKRVAK.GVPAECSM............
TaXTH12.4d ..PEPSQLTKEQ..MRKLRAVQLGYTIYDYCAKARDGGKGPVPPECDMEQY.........
TaXTH12.5a QE.....LDLTR..QQRMRWVQSNYMIYNYCTDPKRFAQ.GVPAECSM............
TaXTH12.5d SPVGTGLTRQQY...AAMRWAQQRHMVYYYCQDFRRDR.SLTP.EC..............
TaXTH12.6a QE.....LDLTR..QQRMRWVQRNYMIYNYCTDPKRVAK.GVPAECSM............
TaXTH12.6d QE.....LDLTR..QQRMRWVQSNYMIYNYCTDPKRFAQ.GVPAECSM............
TaXTH12.7a QE.....LDATA..LDRLQWVQKNYMIYNYCTDTWRFKD.GAPPECATK...........
TaXTH12.7d QE.....LDLTR..QQRMRWVQSNYMIYNYCTDPKRVAK.GLPAECSM............
TaXTH12.8d QE.....LDLTR..QQRMRWVQRNYMIYNYCTDPKRVAQ.GVPAECSM............
2 2

1un1

```
1un1         ..................................
TaXTH1       ..................................
TaXTH2       ..................................
TaXTH3       ..................................
TaXTH4       ..................................
TaXTH5       ..................................
TaXTH6.1a    ESK........KKRRRSSSPLQYSSSMQ......
TaXTH6.1d    ESK........KRRRRSSSPLQYSSSMQ......
TaXTH6.2a    ..................................
TaXTH6.2d    ..................................
TaXTH7.1a    ..................................
TaXTH7.1b    ..................................
TaXTH7.1d    ..................................
TaXTH7.2a    ..................................
TaXTH7.2b    ..................................
TaXTH7.2d    ..................................
TaXTH7.3a    ..................................
TaXTH7.3b    ..................................
TaXTH7.3d    ..................................
TaXTH7.4a    ..................................
TaXTH7.4b    ..................................
TaXTH7.4d    ..................................
TaXTH7.5a    ..................................
TaXTH7.5b    ..................................
TaXTH7.5d    ..................................
TaXTH7.6a    ..................................
TaXTH7.6d    ..................................
TaXTH7.7d    ..................................
TaXTH8.1a    ..................................
TaXTH8.1b    ..................................
TaXTH8.2b    ..................................
TaXTH8.3b    ..................................
TaXTH8.4b    ..................................
TaXTH9.1a    EARSS......DRRRHGKRHRRAGAAL.......
TaXTH9.1b    EARSS......DRRRHGKRHRRAGAADSSL....
TaXTH9.1d    ..................................
TaXTH9.2a    ..................................
TaXTH9.2b    ..................................
TaXTH9.2d    EARSS......DRRRHGKRHRRAGAADSSL....
TaXTH9.3a    ESKAS.....FRSRTTPPPPRPLAVSMMQAD...
TaXTH9.3d    ..................................
TaXTH9.4a    ..................................
TaXTH10.1a   ..................................
TaXTH10.1b   ..................................
TaXTH10.1d   ..................................
TaXTH11.1a   HRLA......LRRRRVGRRPP.....NKADM...
TaXTH11.1b   HRLS......LRRRRVGHRPARPGAANKADM...
TaXTH11.1d   ..................................
TaXTH11.2a   ESKTVVMKARGRGRRGRGSRAGAGARGRAGAASS
TaXTH11.2b   ESKTVVMKTRGRGRRGRGSRAGAGARGRAGAASC
TaXTH11.3a   ..................................
TaXTH11.3b   ..................................
TaXTH11.4b   ..................................
TaXTH12.1a   ..................................
TaXTH12.1b   ..................................
TaXTH12.1d   ..................................
TaXTH12.2a   ..................................
TaXTH12.2b   ..................................
TaXTH12.2d   ..................................
TaXTH12.3a   ..................................
TaXTH12.3b   ..................................
TaXTH12.3d   ..................................
TaXTH12.4a   ..................................
TaXTH12.4b   ..................................
TaXTH12.4d   ..................................
TaXTH12.5a   ..................................
TaXTH12.5d   ..................................
TaXTH12.6a   ..................................
TaXTH12.6d   ..................................
TaXTH12.7a   ..................................
TaXTH12.7d   ..................................
TaXTH12.8d   ..................................
```
