# Supplementary material for: A Surprising Diversity of Xyloglucan Endotransglucosylase/Hydrolase in Wheat: New in Sight to the Roles in Drought Tolerance
Source: Int J Mol Sci. 2023 Jun 8;24(12):9886. doi: 10.3390/ijms24129886 (PMC10297901; doi:10.3390/ijms24129886)
Supplement: Supplementary file 1 [file ijms-24-09886-s001.zip › Supplementary_Figures Several figures cited in the article.pdf]

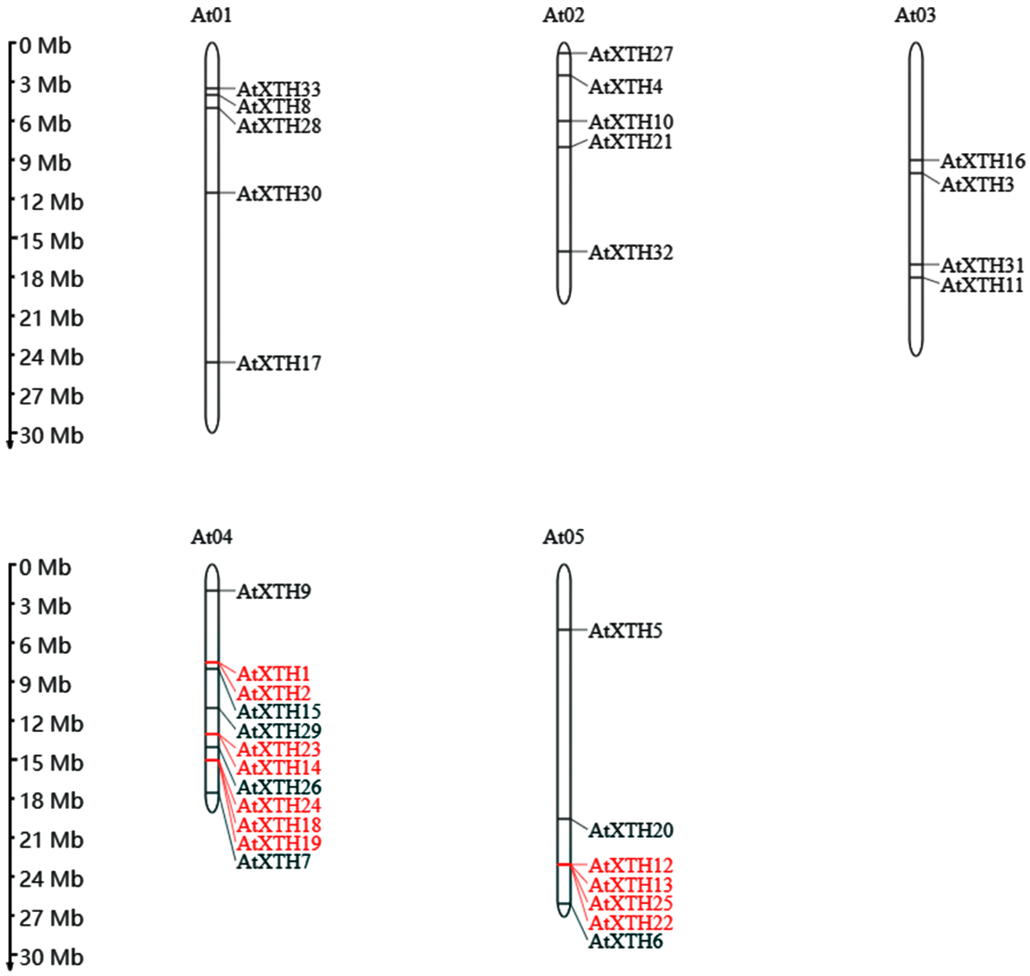

Supplementary figure S1 Distribution of *XTH* genes on *A.thaliana* chromosomes. *AtXTH* genes in red are tandem duplication genes. The number of chromosomes was indicated at the top of each chromosome. The scale on the left is in megabases (Mb).

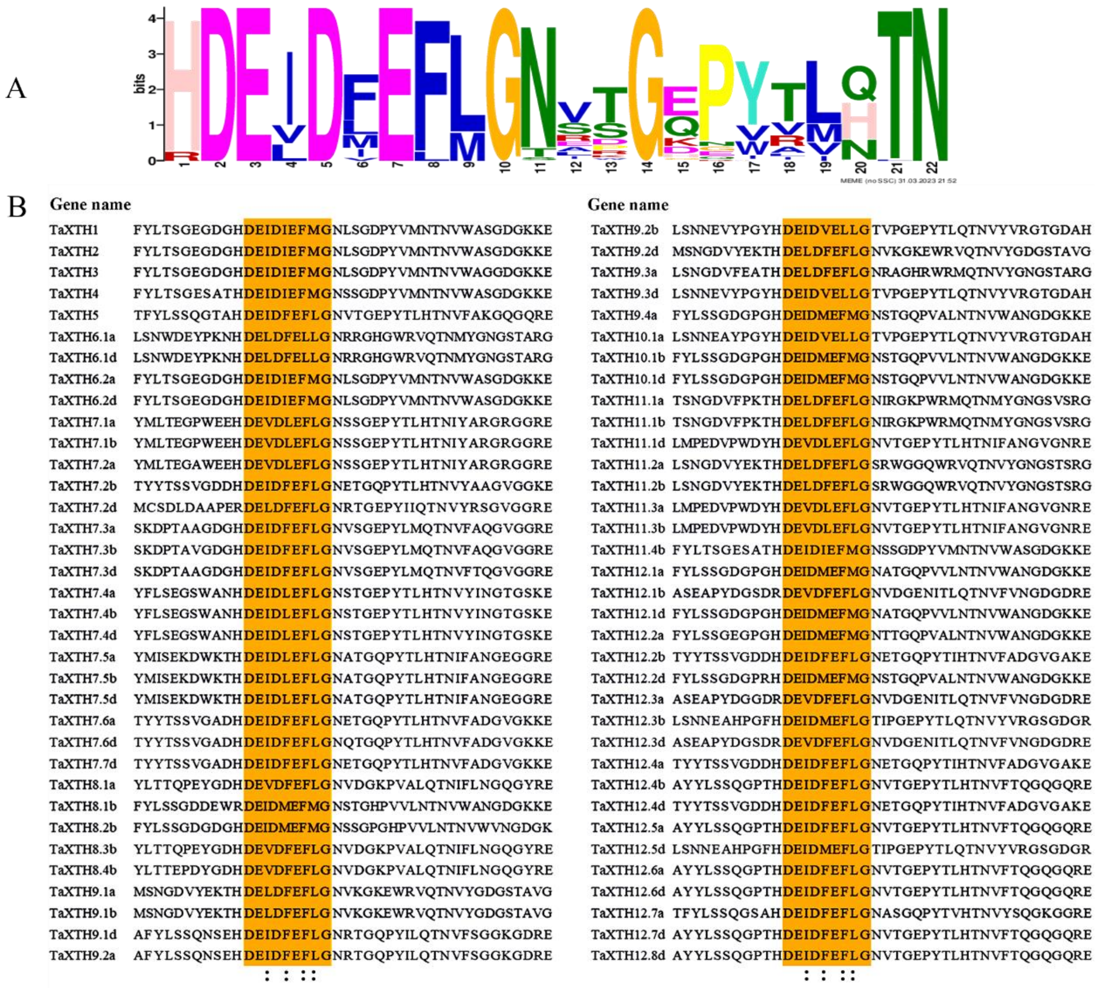

Supplementary figure S2 Conserved protein motifs in wheat XTHs. (A) Catalytically active motifs in the TaXTH proteins. (B) Alignment of the putative-site amino acid residues in TaXTH proteins constructed with the CLUSTALW2. Amino acid residues with the same motif were highlighted with orange shadows.

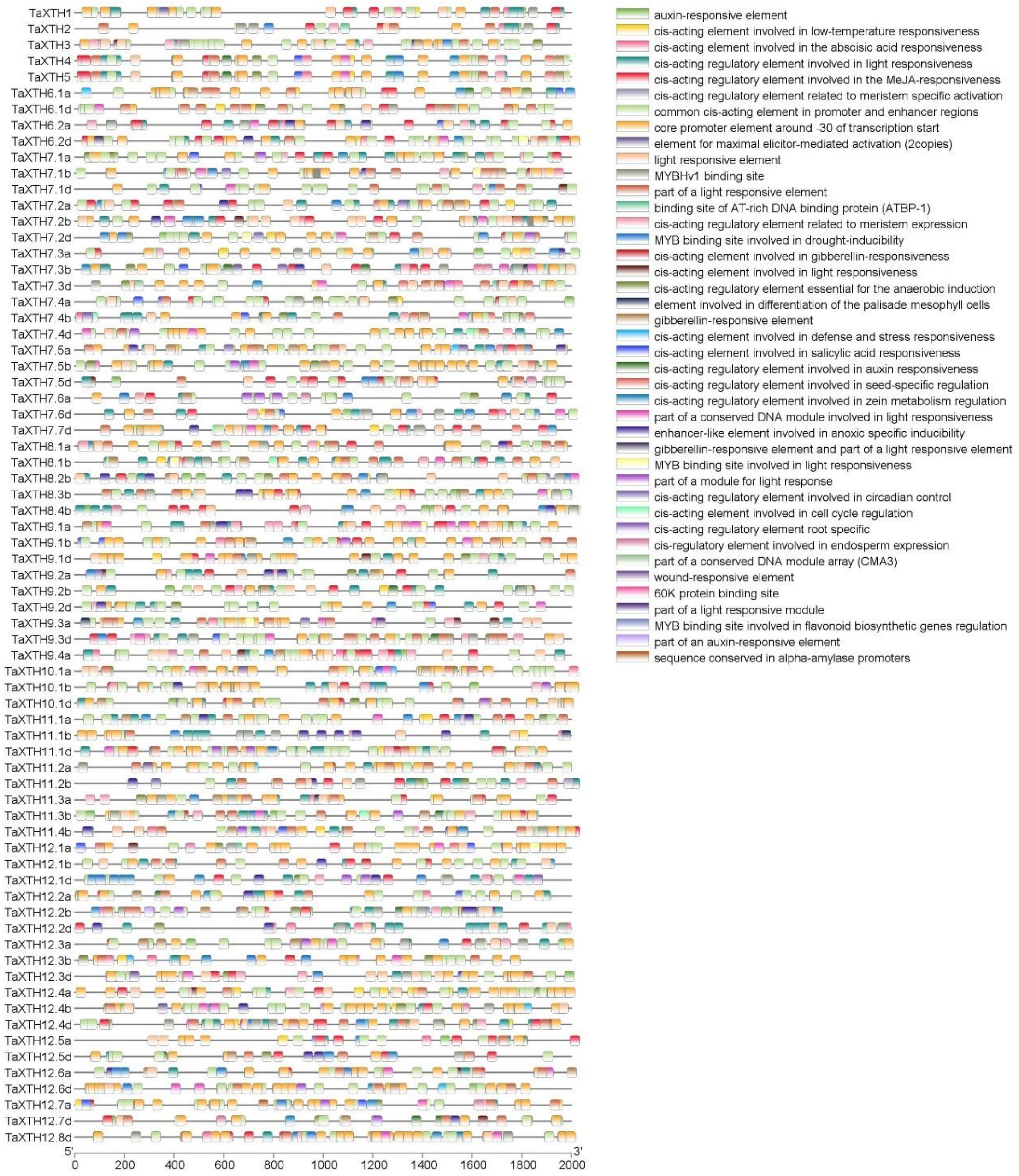

Supplementary Figure S3 Analysis of the binding sites in the promoter regions of *TaXTH* genes. The promoter sequences of 2,000 bp of the *XTH* genes were retrieved from the wheat genome database to analyze the cis-acting regulatory elements (CAREs). PlantCARE (http://bioinformatics.psb.ugent.be/webtools/plantcare/html/) was used for identifying and analyzing the CAREs.

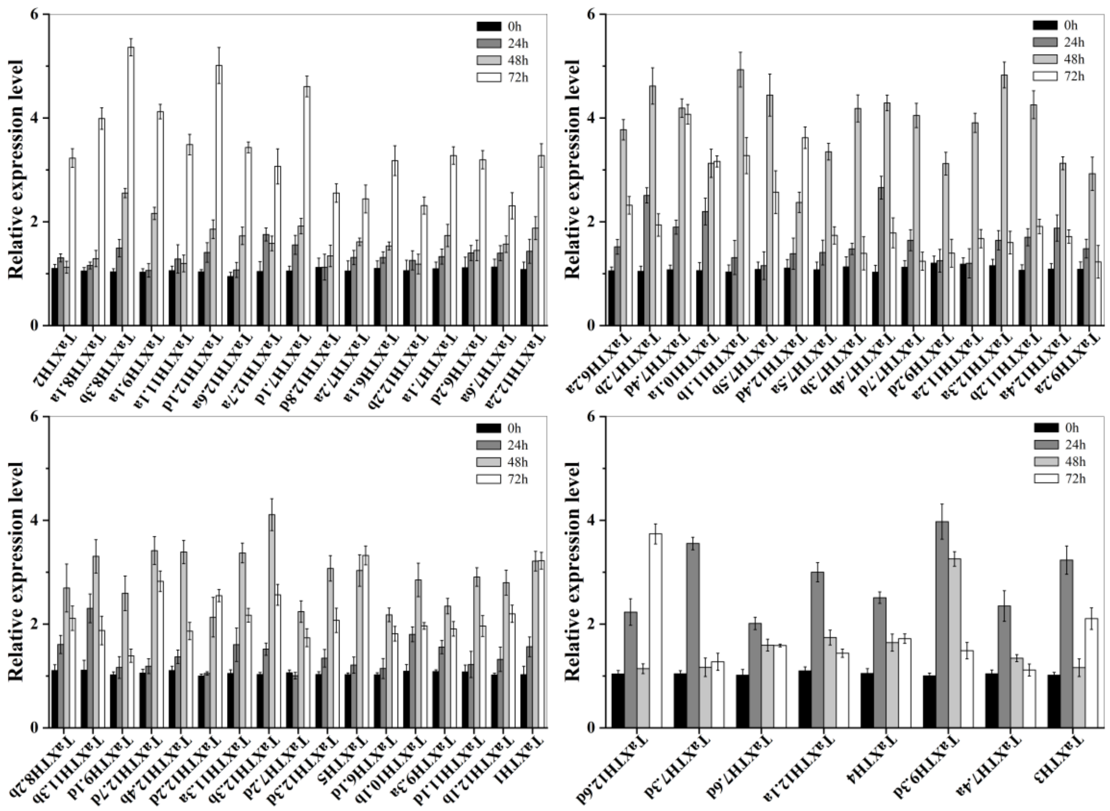

Supplementary Figure S4 The expression of the *TaXTH* gene in the spring wheat line 'Xinchun11' was analyzed using real-time quantitative PCR. The relative expression level of *TaXTH* in wheat was measured after subjecting it to different durations (h) of drought stress with -0.5 MPa D-sorbitol. The 2-day-old germinated wheat seedlings were immersed in -0.5 MPa D-sorbitol and sampled at 0, 24, 48, and 72 hours after treatment to assess the expression of *TaXTH*. Genes exhibiting similar expression patterns are depicted in the same image.

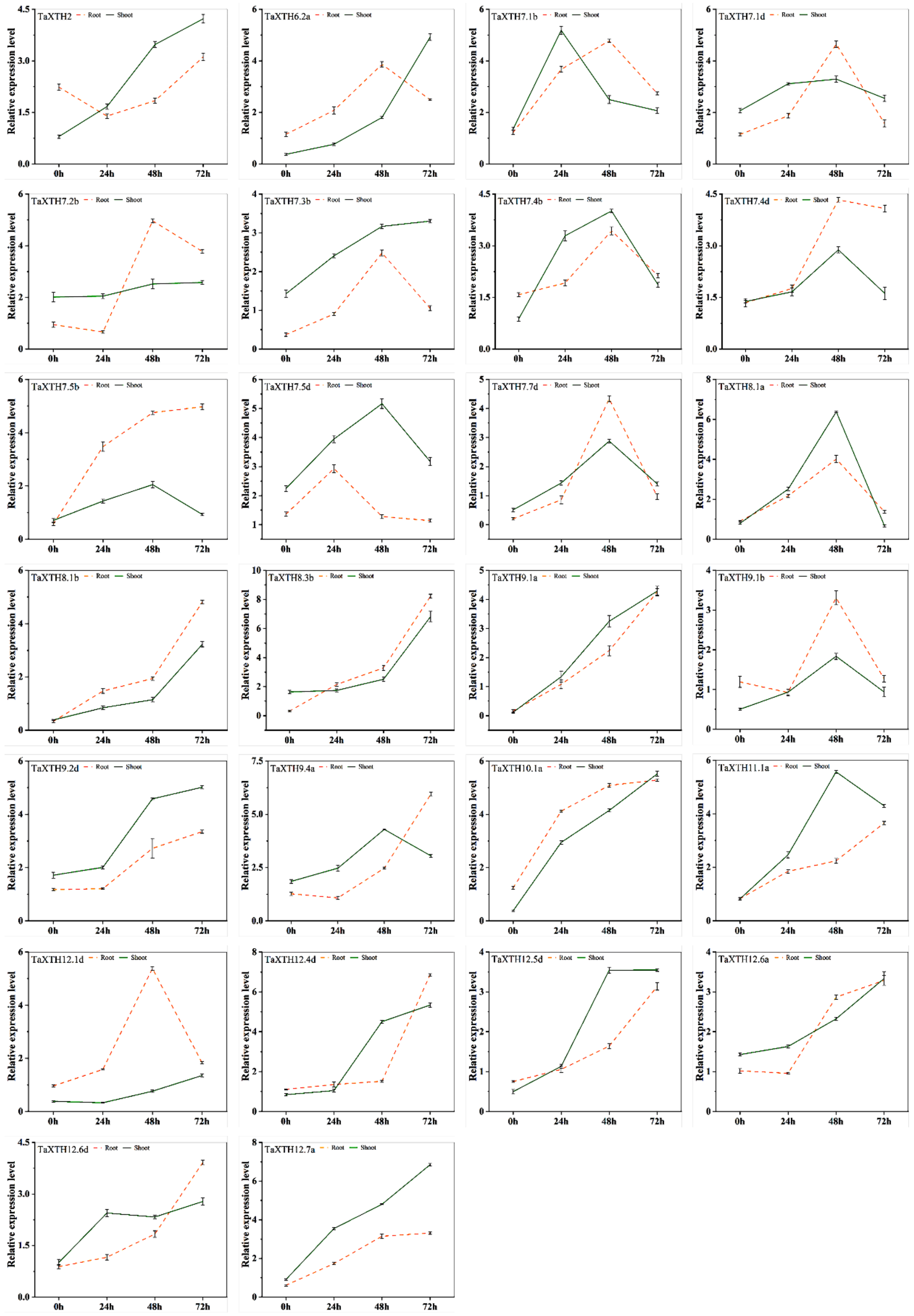

Supplementary Figure S5 qRT-PCR analysis of *TaXTH* genes expressed under drought stress. Samples for expression profiling were collected from wheat at 0, 24, 48, and 72 h post drought stress. Expression profiles were detected by qRT-PCR and normalized to Actin. Note that the relative expression of *TaXTHs* was on a different scale. Orange dashed lines indicate roots, and green solid lines represent shoots. The name of the gene is shown in the upper left corner of each line graph. Results were analyzed in three biological replicates.
